# Supplementary figures and images for: Brain transcriptome of gobies inhabiting natural CO2 seeps reveal acclimation strategies to long‐term acidification
Source: Evol Appl. 2023 Jun 29;16(7):1345–58. doi: 10.1111/eva.13574 (PMC10363848; doi:10.1111/eva.13574)

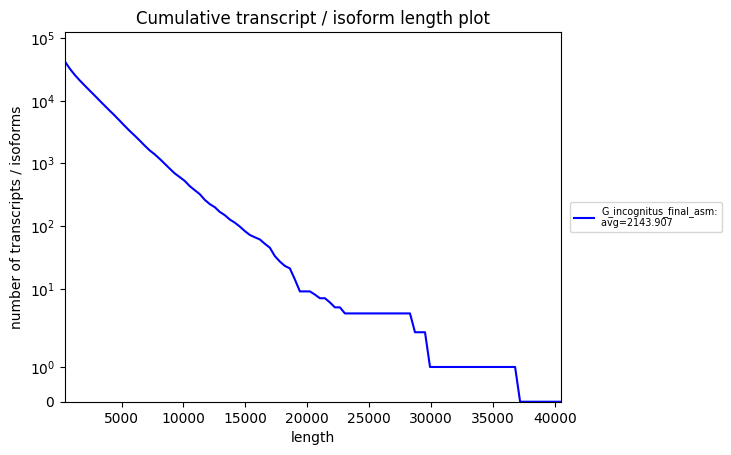

Supplement: Supplementary file 1 — Figure S1. [file EVA-16-1345-s001.png]

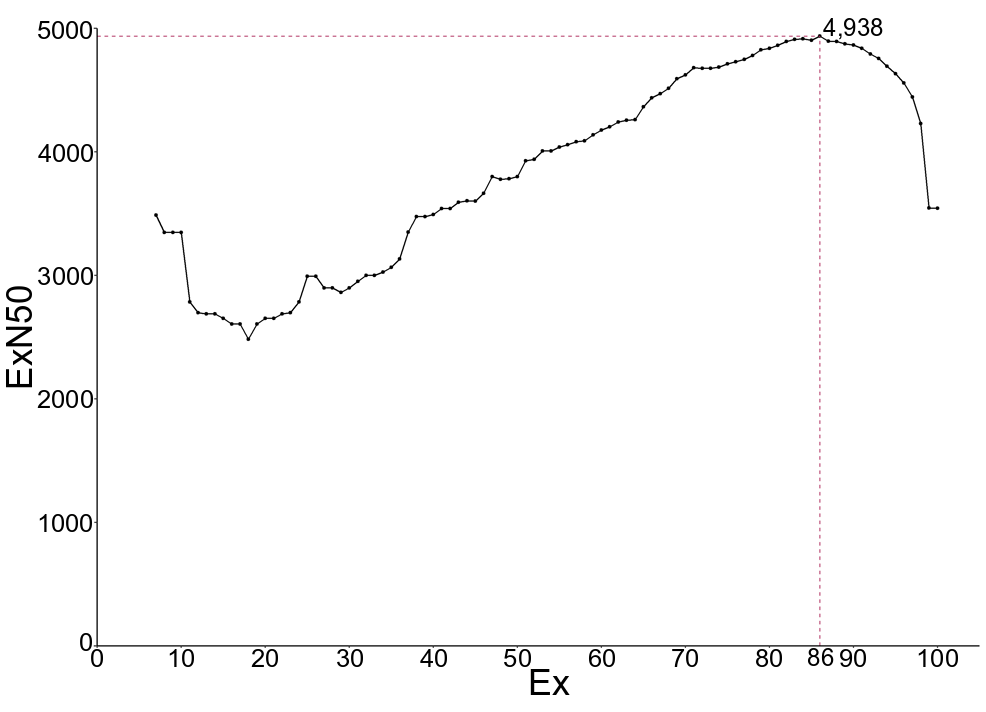

Supplement: Supplementary file 2 — Figure S2. [file EVA-16-1345-s004.png]

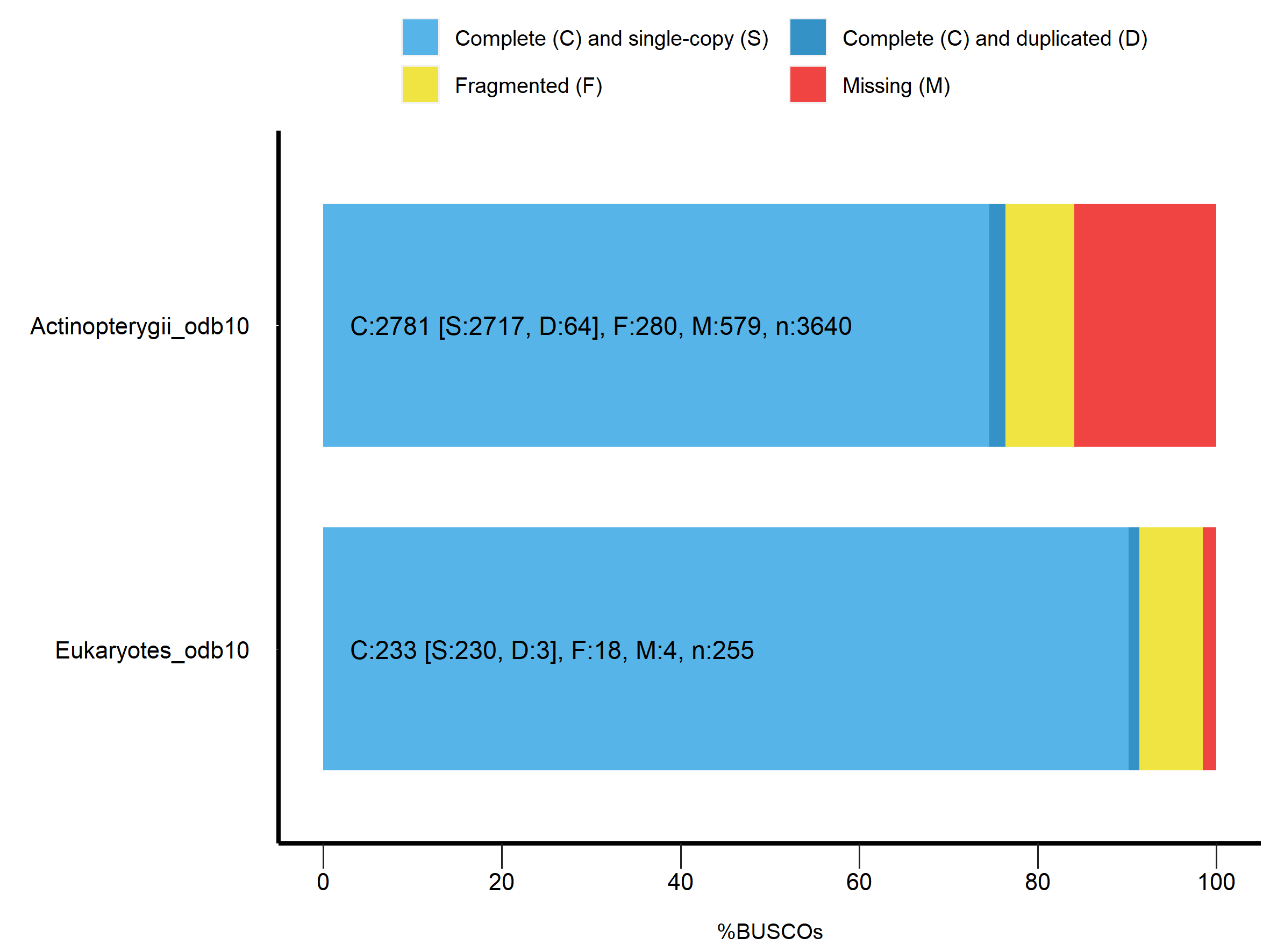

Supplement: Supplementary file 3 — Figure S3. [file EVA-16-1345-s007.png]

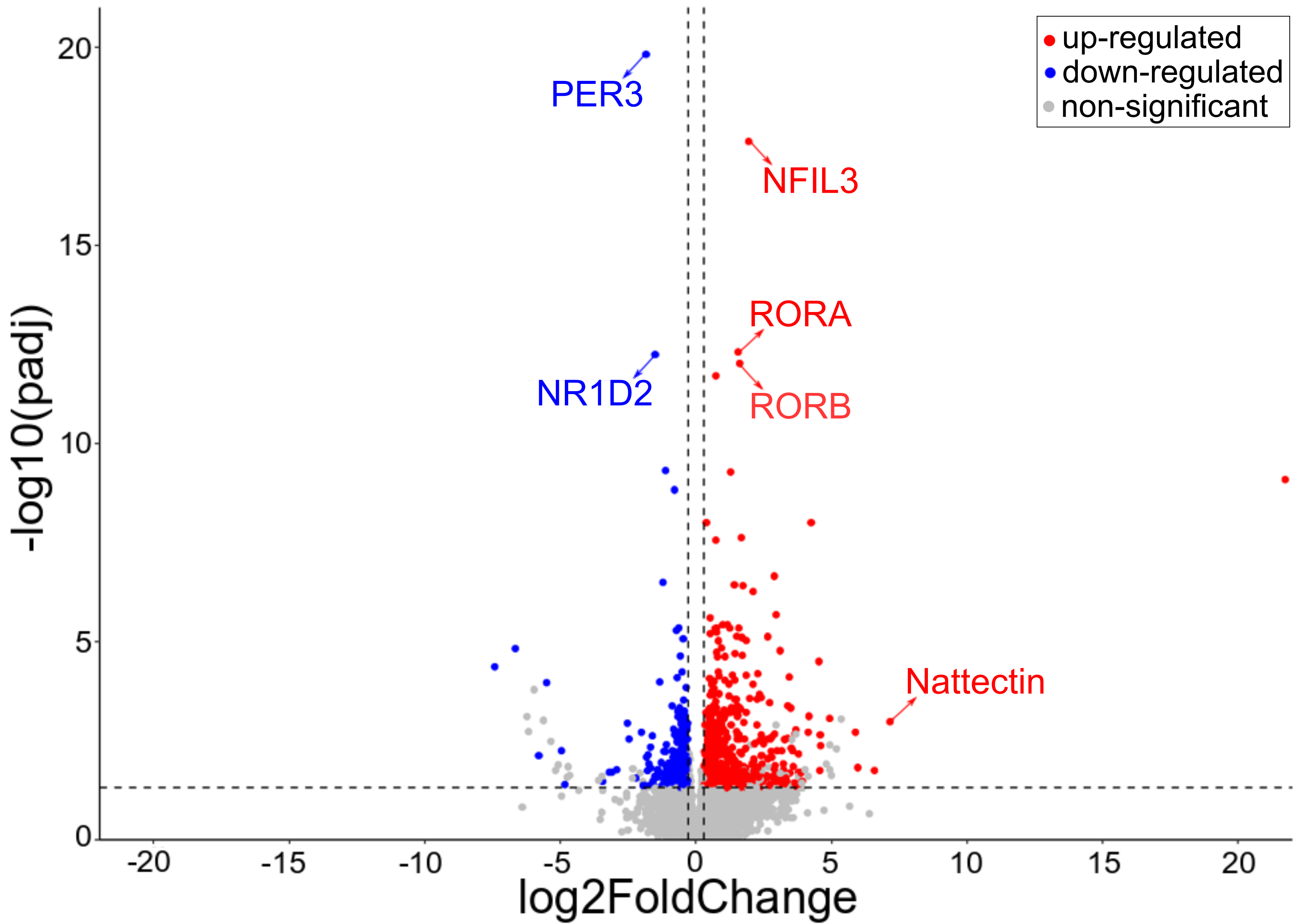

Supplement: Supplementary file 4 — Figure S4. [file EVA-16-1345-s006.png]

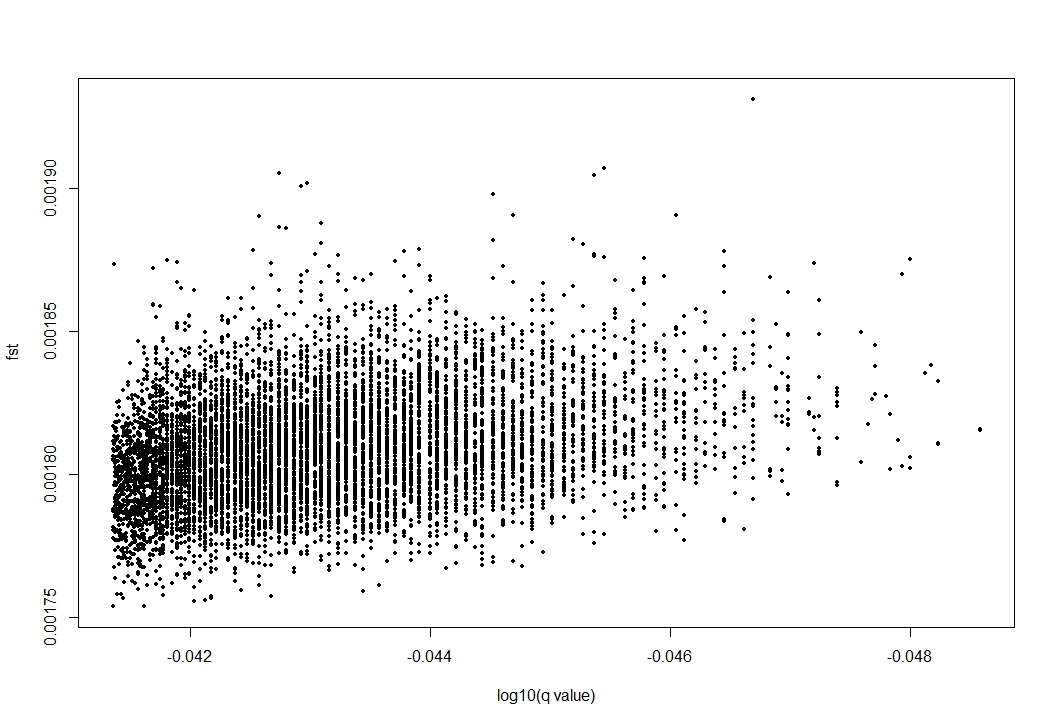

Supplement: Supplementary file 5 — Figure S5. [file EVA-16-1345-s002.jpeg]
