# Supplementary material for: Brain transcriptome of gobies inhabiting natural CO2 seeps reveal acclimation strategies to long‐term acidification
Source: Evol Appl. 2023 Jun 29;16(7):1345–58. doi: 10.1111/eva.13574 (PMC10363848; doi:10.1111/eva.13574)
Supplement: Supplementary file 7 — Appendix S1. [file EVA-16-1345-s003.docx]

**Brain transcriptome of gobies inhabiting natural CO_2_ seeps reveal acclimatisation strategies to long-term acidification**

Sneha Suresh^1^, Alice Mirasole^2^, Timothy Ravasi^3,4^, Salvatrice Vizzini^5,6^, Celia Schunter^1,7*^

^1^Swire Institute of Marine Science, School of Biological Sciences, The University of Hong Kong, Pokfulam Road, Hong Kong SAR

^2^Department of Integrative Marine Ecology, Ischia Marine Centre, Stazione Zoologica Anton Dohrn, via F. Buonocore 42, 80077 Ischia, Naples, Italy

^3^Marine Climate Change Unit, Okinawa Institute of Science and Technology Graduate University, Onna-son, Japan

^4^Australian Research Council Centre of Excellence for Coral Reef Studies, James Cook University, Townsville, Australia

^5^University of Palermo, Department of Earth and Marine Sciences, via Archirafi 22, 90123 Palermo, Italy

^6^CoNISMa, National Inter-University Consortium for Marine Science, Piazzale Flaminio 9, 00196 Roma, Italy

^7^State Key Laboratory of Marine Pollution, City University of Hong Kong, Hong Kong, Hong Kong SAR, China

*Corresponding author

Correspondence: [celiaschunter@gmail.com](about:blank)

**SUPPLEMENTARY FIGURES**

**Supplemenary Figure 1:**

**Figure S1:** Cumulative length distribution of all assembled transcripts in the *de novo* transcriptome. The *de novo* assembled transcriptome consisting of 43,349 contigs (transcripts) has an average length of 2,143.9 bp with an N50 value of 3,545, L50 value of 7,998 and 61% of the contigs over 1Kbp in length.

**Supplemenary Figure 2:**

**Figure S2:** Expression-informed N50 (ExN50) calculated across different expression percentiles (Ex) using the TMM normalised counts. The highest ExN50 value (4,938 bp) was obtained when including transcripts representing 86% of total normalised expression indicating good coverage of longer transcripts in the *de novo* assembly.

**Supplemenary Figure 3:**

**Figure S3:** BUSCO assessment of assembly completeness using the Actinopterygii_odb10 and Eukaryota_odb10 core gene dataset. The analyses revealed that the *de novo* assembled transcriptome had a high gene completeness with 84% and 98% of genes from the Actinopterygii and Eukaryota library recovered respectively

**Supplemenary Figure 4:**

**Figure S4:** Volcano plot showing log2 fold change in transcript expression levels between individuals from control and CO_2_ seep (LPH) sites on the x-axis (positive values are up-regulated and negative values are down-regulated in fish from the CO_2_ seeps sites) and the negative log10 of FDR corrected p-values on the y-axis. Transcripts that are not significantly DE are in grey, transcripts that are significantly DE are in red (up-regulated) and blue (downregulated).

**Supplemenary Figure 5:**

**Figure S5:** Detection of putative outlier loci between the samples from the CO_2_ seep and control sites using the Bayesian based BayeScan program. The graph represents the F_ST_ values against the corresponding log10(FDR corrected p-value(q value)) for each loci. There were no significant outlier loci detected between the samples from the CO_2_ seep and control sites.
